# Access to oral care is a human rights issue: a community action report from the Downtown Eastside of Vancouver, Canada

**DOI:** 10.1186/s12954-022-00626-4

**Published:** 2022-05-02

**Authors:** Ehsan Jozaghi, Vandu, Russ Maynard, Yasaman Khoshnoudian, Mario A. Brondani

**Affiliations:** 1 Faculty of Dentistry, 116/2199 Wesbrook Mall, University of British Columbia, Vancouver, BC V6T 1Z3 Canada; 2 Vancouver Area Network of Drug Users (VANDU), 380 East Hastings St, Vancouver, BC V6A 1P4 Canada; 3 PHS Community Services Society, 9 E Hastings St, Vancouver, BC V6A 1M9 Canada

**Keywords:** VANDU, Oral health, Underserved, Marginalized, Dentistry, People who use drugs, Subsidized, Downtown Eastside, Access to oral care, Stigma

## Abstract

To offer a critical reflection on an impoverished neighborhood in Vancouver, Canada, and their access to oral health care. A review of how a lack of publicly funded oral health care affects the most vulnerable, uninsured, and underserved citizens is performed. Personal and professional accounts on how entrepreneurial innovations of not-for-profit organizations can help to close the gap in access to oral health care are offered using the Vancouver Area Network of drug users (VANDU) and the PHS Community Services Society as case studies in British Columbia. Despite the efforts put forward by not-for-profit organizations such as the VANDU and the PHS Community Services Society, a national oral health care plan is warranted though still not a political imperative. Underserved citizens have a right to oral health care that is compassionate, collaborative, accessible, and affordable.

## Background

Every year, thousands of underserved citizens experience unnecessary suffering from oral conditions, mainly dental decay and periodontal diseases. Although largely preventable, these diseases are a threat to general health; they are a silent epidemic [1].

There are a number of chronic illnesses that are linked to poor oral health, including heart disease [2], stroke [3], type 2 diabetes [4], aspiration pneumonia [5], and possibly Alzheimer’s disease [6]. Moreover, there are reports on the direct link between timely oral health care and improved self-esteem [7], better dietary intake [8], and increased overall mental health and school performance [9].

Previous studies have also established links between oral health morbidity (dental caries, periodontal disease) and mortality (tooth loss) compounded by various factors including racism [10], lower income [11], rural residency [12], and lack of dental insurance [13]. While oral health disparities are prevalent across the globe [14], the financial impact of COVID-19 (SARS-CoV-2 novel coronavirus) has exacerbated the oral health inequities in advanced economies, as millions of citizens have lost employer-sponsored dental insurance [15].

For many ordinary citizen, accessing an oral health care provider for proper care has been inaccessible and expensive even before the pandemic [16]. Although certain provinces offer some form of government funded dental insurance [16, 17], public oral health care is not an integral part of the health care system in many nations as it is in other countries, such as Brazil and Sweden [18]. In Canada, for example, for those who can afford these costs, more than one-third of the 13 billion dollars spend annually in oral health care comes out of their pockets [26]; only about 6% of this expense comes from government-sponsored insurances—dental plans—at federal, provincial, or municipal levels [26]. It becomes clear that many underserved populations need a national oral health care plan based on principles of human rights and access to health care. Only recently has there been some interest in a publicly funded oral health care system in Canada, by government officials based on income [19]. Therefore, in this commentary that is driven by two prominent community organizations, we report on how a lack of publicly funded oral health care affects the most vulnerable, uninsured, and underserved citizens. While using the Downtown Eastside (DTES) of Vancouver in British Columbia as a context for this conversation, we also present the perspective of practitioners and how entrepreneurial innovations in the area of not-for-profit organizations have attempted to close the gap in access to oral health care. Ultimately, we argue that a national oral health care plan for underserved citizens is a human rights issue because oral health and dental disease are linked to many major health concerns with significant personal and societal implications.

## VANDU activisms on oral health

Oral health has been an integral part of activism for the Vancouver Area Network of Drug User (VANDU) members who have fought for proper harm reduction supplies and safe spaces for people who use drugs [20]. For example, in early 2000, VANDU members distributed heat resistant pipes for people who smoke drugs (PWSDs) to prevent injury to their mouths, lips, tongue, and other oral structures [20]. At the same time, to prevent oral abscess and injury, VANDU members formed educational groups to teach PWSDs to use proper equipment (e.g., mouth pieces and brass screens) when smoking drugs to reduce blood borne infections (HIV, and Hepatitis C (HCV)) and prevent oral sores and blisters [20]. To reduce the risk of blood borne infections and overdose deaths among PWSDs, VANDU established the first unsanctioned smoking facility in the DTES of Vancouver [21]. The unsanctioned smoking facility has been studied by external researchers and has been shown to not only save tax payers dollars by preventing HCV cases among PWSDs [22], but to also decrease health-related harms within these underserved population [21].

However, access to oral care for VANDU members cannot be simply linked to harm reduction in the context of PWSDs, because access to care is acutely lacking within the criminalized and underserved drug-using population of the DTES. Many PWUDs in the DTES report high levels of dental caries and come to VANDU for information related to oral health support. Although there is no strong evidence to establish a direct link between opioid substitution treatment therapy, such as methadone usage, and increased dental caries [23], many VANDU members report poor dental care and high dental caries linked to higher sugar mixed with many opioid substitution therapies, lack of proper daily oral hygiene and nutritional diet. At the same time, many VANDU members have lost family members and loved ones to chronic illnesses linked to lack of access to oral health care, including, but not limited to, endocarditis and heart disease linked to oral abscesses; many members are highly traumatized.

In addition, many VANDU members have raised concerns about the stigma and discrimination they face when attempting to access much needed oral health care, and a lack of knowledge from oral care professionals when interacting with PWUDs. Additionally, there are also many incidents of refused oral health treatments for PWUDs. Many VANDU members have also felt pressured by dentists to have their tooth extracted as the only option, due to a lack of or limited dental coverage. As a result, many VANDU members have many missing teeth at an early age, which affects their confidence and self-esteem. Tooth loss also limits their ability to consume solid foods, which hinders their healthy food choices and overall well-being.

## The Portland Community Dental Clinic in the DTES community

To begin to address some of the many issues described above among the underserved community members of the DTES, PHS Community Services Society established a not-for-profit dental clinic in the DTES in 2001. PHS was established in the early 1990s in order to provide housing, health care, and social services to people euphemistically referred to as “hard to house” [24]. PHS was an important originator of what is now referred to as Housing First, an approach to housing and services based on the philosophy of providing services in a relevant, low-barrier, and accessible way, where the underserved populations’ basic human rights, dignity, and respect are met in their community.

Since its inception, the community dental clinic has served 1448 patients annually. The clinic currently provide support to many residents of the DTES by providing oral health care to 8–12 patients per day on average. Two full-time staff dentists provide comprehensive oral health care to individuals on income assistance, job training, and other pre-employment programs. The clinic is currently welcoming many clients regardless of their socioeconomic background, lifestyle histories, or medical conditions based on the principles of dignity and trauma-informed care.

The clinic’s focus has been to provide oral health care that is comprehensive, which also includes restorative and preventative dentistry. Annually, the clinic also provides extractions, fillings, root canals, dentures, and even crowns. What is more unique is the commitment to provide oral care in a very person-centered and thoughtful approach to a population that is often very unfamiliar with dentistry and oral health yet is at high risk of oral health complications.

The clinic has also been an affiliated teaching site for University of British Columbia (UBC)'s dentistry program, where senior 4th year students perform clinical work under the supervision of instructors. Also, the clinic provides delivery of care by UBC’s General Practitioner Residents also affiliate with the Faculty of Dentistry.

## Summative points

As discussed above, oral health is linked to general health and well-being—the mouth belongs to the body. Also discussed was the fact that many Canadians, including those living in the DTES, face various barriers when attempting to obtain oral health care. One of these barriers is a lack of insurance or disposable income [25]. Not surprisingly, many patients are avoiding dentist due to affordability issues and are potentially at the greater risk of many preventable diseases. For example, 1 in every 5 Canadians avoid visiting a dentist because they cannot afford the costs [17]. The PHS clinic in the DTES has demonstrated how not-for-profit dentistry is increasingly filling the gap in oral health care for underserved citizens. Not-for-profit oral care has also shown to benefit the health care system as it provides educational opportunities to those involved via partnership, such as the UBC dentistry program, for many future dentist unfamiliar with social justice and health inequity issues.

The Canadian Association of Emergency Physicians supports the expansion of publicly funded and delivered oral health care, since patients covered by Medicare make emergency room visits for their dental-related problems, adding further strain on Emergency Departments [27, 28]. Similarly, one of the main Canadian political parties has recently advocated for a federally funded dental coverage to uninsured individuals with a household income of less than $70,000 a year [29]. This advocacy prompted the Office of the Parliamentary Budget Officer to generate a report estimating the cost of establishing a Federal dental care program for uninsured Canadians with a household income of less than $90,000 [30].

It seems that the idea of a national oral health care plan has gained momentum in some countries. Aside from the logistics of implementing such a plan, the (in)ability to pay for oral health care does not tell the whole story. The reasons behind these experiences can be due to the low payment most government-sponsored dental plans offer to providers, who then try to offset the substantial overhead many endure. But worse, it can be due to stigma and the discrimination members of underserved communities face when visiting a dental provider’s office [32–34, 37, 38] as experienced by the DTES residents and VANDU members; they also experience many other health inequities.

Therefore, a national oral health care plan is a human rights issue because healthy mouths are linked to a healthy body and poor access to dental services is having a significant impact on many underserved citizens [39]. Similar to other previous government and non-profit reports, we urgently call on governments and health agencies to ensure that oral health care and dental treatment is affordable and available to all people experiencing health disparities, homelessness, or poverty [39]. Similarly, we urge the dental schools around the world to focus on the training and education of future oral health care providers on the social determinants of health, social responsibility, trauma and violence informed care, cultural safety and humility, and person-centered care, so that stigma and discrimination is eliminated in dental medicine and oral care [40, 41]. Underserved citizens have a right to oral health care that is compassionate, accessible, collaborative, and affordable.

## Data Availability

Not applicable.

## References

[1] Benjamin RM (2010). Oral health: the silent epidemic. Public Health Rep.

[2] Kim K, Choi S, Chang J, Kim SM, Kim SJ, Kim RJ, Cho HJ, Park SM (2019). Severity of dental caries and risk of coronary heart disease in middle-aged men and women: A population-based cohort study of Korean adults, 2002–2013. Sci Rep.

[3] Chang Y, Woo HG, Lee JS, Song TJ (2021). Better oral hygiene is associated with lower risk of stroke. J Periodontol.

[4] Leite RS, Marlow NM, Fernandes JK, Hermayer K (2013). Oral health and type 2 diabetes. Am J Med Sci.

[5] Müller F (2015). Oral hygiene reduces the mortality from aspiration pneumonia in frail elders. J Dent Res.

[6] Leira Y, Dominguez C, Seoane J, Seoane-Romero J, Pías-Peleteiro JM, Takkouche B, Blanco J, Aldrey JM (2017). Is periodontal disease associated with Alzheimer's disease? A systematic review with meta-analysis. Neuroepidemiology.

[7] Kenealy PM, Kingdon A, Richmond S, Shaw WC (2007). The Cardiff dental study: a 20-year critical evaluation of the psychological health gain from orthodontic treatment. Br J Health Psychol.

[8] Piesman M, Kozarek RA, Brandabur JJ, Pleskow DK, Chuttani R, Eysselein VE, Silverman WB, Vargo JJ, Waxman I, Catalano MF, Baron TH (2009). Improved oral intake after palliative duodenal stenting for malignant obstruction: a prospective multicenter clinical trial. ACG.

[9] Guarnizo-Herreño CC, Wehby GL (2012). Children's dental health, school performance, and psychosocial well-being. J Pediatr.

[10] Hedges J, Haag D, Paradies Y, Jamieson L (2021). Racism and oral health inequities among Indigenous Australians. Community Dent Health.

[11] Williams S, Wei L, Griffin SO, Thornton-Evans G (2021). Untreated caries among US working-aged adults and association with reporting need for oral health care. J Am Dent Assoc.

[12] Hamano T, Takeda M, Tominaga K, Sundquist K, Nabika T (2017). Is accessibility to dental care facilities in rural areas associated with number of teeth in elderly residents?. Int J Environ Res Public Health.

[13] Basir L, Rasteh B, Montazeri A, Araban M (2017). Four-level evaluation of health promotion intervention for preventing early childhood caries: a randomized controlled trial. BMC Public Health.

[14] Costa SM, Martins CC, Bonfim MD, Zina LG, Paiva SM, Pordeus IA, Abreu MH (2012). A systematic review of socioeconomic indicators and dental caries in adults. Int J Environ Res Public Health.

[15] Choi SE, Simon L, Riedy CA, Barrow JR (2021). Modeling the impact of COVID-19 on dental insurance coverage and utilization. J Dent Res.

[16] Flaer PJ, Younis MZ, Benjamin PL, Al-Hajeri M (2011). The political culture of healthcare: why substantial dental care in Canada is covered by government insurance only in Québec—lessons for the United States?. Br Dent J.

[17] Canadian Academy of Health Sciences. Improving access to oral health care for vulnerable people living in Canada. 2014 [cited 2021 Oct 10th]. https://cahs-acss.ca/wp-content/uploads/2015/07/Access_to_Oral_Care_FINAL_REPORT_EN.pdf.

[18] Celeste RK, Nadanovsky P, Fritzell J (2011). Trends in socioeconomic disparities in the utilization of dental care in Brazil and Sweden. Scand J Public Health.

[19] Ritchie H. Liberals introduce a new budget with universal dental and safe supply funding. 2021[cited 2021 Oct 10th]. https://www.yukon-news.com/news/liberals-introduce-new-budget-with-universal-dental-and-safe-supply-funding/?_cldee=c2FyYWhibGF3YXR0QGdtYWlsLmNvbQ%3d%3d&recipientid=contact-36d31e51a2d6ea11a813000d3af4a4ca-af334bdef7ad43ad9924a61a0e55664e&esid=2cf362f7-16b7-eb11-8236-000d3a09cf66.

[20] Jozaghi E, Lampkin H, Andresen MA (2016). Peer-engagement and its role in reducing the risky behavior among crack and methamphetamine smokers of the Downtown Eastside community of Vancouver, Canada. Harm Reduct J.

[21] McNeil R, Kerr T, Lampkin H, Small W (2015). “We need somewhere to smoke crack”: an ethnographic study of an unsanctioned safer smoking room in Vancouver, Canada. Int J Drug Policy.

[22] Jozaghi E (2014). A cost-benefit/cost-effectiveness analysis of an unsanctioned supervised smoking facility in the Downtown Eastside of Vancouver, Canada. Harm Reduct J.

[23] Tripathee S, Akbar T, Richards D, Themessl-Huber M, Freeman R (2013). The relationship between sugar-containing methadone and dental caries: a systematic review. Health Educ J.

[24] Evans L, Strathdee SA (2006). A roof is not enough: Unstable housing, vulnerability to HIV infection and the plight of the SRO. Int J Drug Policy.

[25] Thompson B, Cooney P, Lawrence H, Ravaghi V, Quiñonez C (2014). Cost as a barrier to accessing dental care: findings from a C anadian population-based study. J Public Health Dent.

[26] Canadian Dental Association. Dental health services in Canada. 2017 [cited 2021 Oct 10th]; https://www.cda-adc.ca/stateoforalhealth/servicescanada/.

[27] Brondani M, Ahmad SH (2017). The 1% of emergency room visits for non-traumatic dental conditions in British Columbia: Misconceptions about the numbers. Can J Public Health.

[28] Sheikh H. CAEP position statement on dental care in Canada. [Internet]. 2019 July [cited 2021 Oct 10th]. https://caep.ca/wp-content/uploads/2019/09/CAEP-Dental-Care-Statement-CLEAN-VERSION-July-2019-002.pdf.

[29] Gatehouse J. NDP’s dental plan seen as smart policy-cbc.ca [Internet]. 2019 Fall [cited 2021 Oct 10th]. https://www.cbc.ca/news/politics/ndp-dental-plan-fact-check-1.5289005.

[30] Sourang D, Worswick A*. Cost estimate of a federal dental care program for uninsured Canadians.* Office of the Parliamentary Budget Officer. 2020 [cited 2021Oct 10^th^]. https://www.pbo-dpb.gc.ca/en/blog/news/RP-2021-028-M--cost-estimate-federal-dental-care-program-uninsured-canadians--estimation-couts-lies-un-regime-soins-dentaires-federal-destines-tous-canadiens-non-assures.

[31] Macdonald ME, Keboa MT, Nurelhuda NM, Lawrence HP, Carnevale F, McNally M, Singhal S, Ka K, Nicolau B (2019). The oral health of refugees and asylum seekers in Canada: a mixed methods study protocol. Int J Environ Res Public Health.

[32] Mago A, MacEntee MI, Brondani M, Frankish J (2018). Anxiety and anger of homeless people coping with dental care. Commun Dent Oral Epidemiol.

[33] Brondani MA, Alan R, Donnelly L (2017). Stigma of addiction and mental illness in healthcare: the case of patients’ experiences in dental settings. PLoS ONE.

[34] Brondani MA, Phillips JC, Kerston RP, Moniri NR (2016). Stigma around HIV in dental care: patients’ experiences. J Can Dent Assoc.

[35] Ashworth A (2018). Understanding the factors influencing the Aboriginal health care experience. Can J Dent Hygiene.

[36] Katryn Blanchard A, Wang X, El-Gabalawy H, Tan Q, Orr P, Elias B, Rawsthorne P, Hart D, Chubey S, Bernstein C (2012). Oral health in a First Nations and a non-Aboriginal population in Manitoba. Int J Circumpolar Health.

[37] Brondani MA (2014). Patients with poor oral health status received little dental care and patients at the terminal stage of their lives received comprehensive dental treatment. J Evid Based Dent Pract.

[38] Salmasi A, Harrison R, Brondani MA (2015). They stole her teeth! An exploration of adults with developmental disability experiences with dental care. Spec Care Dent.

[39] Groundswell MB. Healthy mouths: a peer-led health audit on the oral health of people experiencing homelessness. 2017. Groundswell-Healthy-Mouths-Report-Full-Report-Web.pdf.

[40] Brondani M, Pattanaporn K, Aleksejūnienė J (2015). How can dental public health competencies be addressed at the undergraduate level?. J Public Health Dent.

[41] Brondani MA (2012). Teaching social responsibility through community service-learning in predoctoral dental education. J Dent Educ.

